# Elevated lipoprotein(a) and progression of aortic stenosis measured by Doppler echocardiography: A population‐based cohort study

**DOI:** 10.1111/joim.20095

**Published:** 2025-05-01

**Authors:** Jonas Brinck, Karin Littmann, Daniel Eriksson Hogling, Linnea Widman, Kenneth Caidahl, Maria Eriksson, Jonas Johnson, Karolina Szummer, Magnus Bäck

**Affiliations:** ^1^ Unit of Endocrinology, Theme Inflammation and Ageing Karolinska University Hospital Stockholm Sweden; ^2^ Department of Medicine Huddinge Karolinska Institutet Stockholm Sweden; ^3^ Department of Molecular Medicine and Surgery Karolinska Institutet Stockholm Sweden; ^4^ Unit of Clinical Physiology Heart and Vascular Center Karolinska University Hospital Stockholm Sweden; ^5^ Department of Clinical Science Intervention and Technology, Karolinska Institutet Stockholm Sweden; ^6^ Department of Cardiology Heart and Vascular Center Karolinska University Hospital Stockholm Sweden; ^7^ Department of Medicine Solna Karolinska Institutet Stockholm Sweden

**Keywords:** calcified aortic valve disease, Doppler echocardiography, lipoprotein(a), peak aortic jet velocity

**Abbreviation**
AS: aortic stenosis
Lp(a): lipoprotein(a)

Elevated lipoprotein(a) (Lp(a)) is a dyslipoproteinaemia that causes atherosclerotic cardiovascular disease and calcified aortic valve stenosis (AS) [1, 2]. AS develops over decades and can lead to valve obstruction requiring an aortic valve procedure. Both genetic variations in the LPA gene and an elevated plasma Lp(a) level are associated with an increased incidence of AS [3, 4]. Measurement of peak aortic jet velocity (*V*_max_; m/s) with Doppler echocardiography is used to assess and monitor AS in clinical routine. In the ASTRONOMER trial, a limited number of participants (*n* = 220) with mild‐to‐moderate AS (initial *V*_max_ ≥ 2.5 m/s) who underwent repeated echocardiography exhibited faster *V*_max_ progression with elevated Lp(a) [5, 6, 7]. Similar faster haemodynamic progression at higher Lp(a) levels was reported in 129 subjects with AS (initial *V*_max_ ≥ 2.0 m/s) from the SATIRE trial and Ring of Fire study [8]. However, the impact of Lp(a) on AS progression in an unselected population has remained unexplored. The aim of the present study was to determine the effect of elevated plasma Lp(a) on AS progression measured as *V*_max_ by repeated Doppler echocardiography in a large cohort with and without AS at any stage.

We performed an observational retrospective cohort study by linking two databases of individuals who had had their plasma Lp(a) level measured 2003–2017 (*n* = 23,398) and who had ≥2 Doppler echocardiography *V*_max_ measurements with ≥6 months of interval 2003–2018 (*n* = 9889) in the clinical routine in Region Stockholm, Sweden (Fig. S1). Lp(a) was analysed using accredited laboratory methods, reporting values in nmol/L or mg/dL as previously described [9]. Participants were subdivided into three Lp(a) strata defined as low (<70 nmol/L or <30 mg/dL), intermediate (70–169 nmol/L or 30–69 mg/dL) and high (≥170 nmol/L or ≥70 mg/dL). Transthoracic Doppler echocardiography was performed at Karolinska University Hospital using standard equipment. The current European Society of Cardiology guidelines recommendation on AS management in relation to AS progression is based on the change in *V*_max_, measured in m/s/year, which was calculated in the present study as the difference between the first and the last Doppler measurement divided by the time interval. Individuals who underwent aortic valve replacement (AVR) were excluded if the procedure was performed before the first echocardiography and censored if undergoing AVR during the observational period. The individuals’ cardiovascular status was specified by the International Classification of Diseases diagnoses. Differences between individuals in Lp(a) strata were compared using the Kruskal–Wallis test, Dunn's post hoc test and unbalanced adjusted analysis of covariance with ranked transformed data. The study was approved by the Ethical Review Authority.

A total of 694 subjects (average 3.5 Doppler measurements/subject) met the study criteria and were allocated to the low (*n* = 434), intermediate (*n* = 125) and high (*n* = 135) Lp(a) subgroups (Table S1). The mean age in the groups was 51, 50 and 58 years, and the proportion of women was 37%, 43% and 39%, respectively, at the timepoint of the first Doppler measurement. The initial median *V*_max_ and the median follow‐up time of *V*_max_ progression were similar between subgroups with 1.42 m/s and 3.7 years (low), 1.44 m/s and 2.9 years (intermediate), and 1.42 m/s and 3.0 years (high) (*p* = 0.82 and 0.35). The median (25th–75th percentile) progression rates were 0.01 (−0.04 to 0.07), 0.02 (−0.03 to 0.14) and 0.03 (−0.02 to 0.12) m/s/year, respectively (*p* = 0.013) (Fig. 1). The difference in *V*_max_ progression between Lp(a) strata remained significant after adjustment for age, sex and baseline *V*_max_ (*p* = 0.027). The *V*_max_ progression was significantly higher in the intermediate (*p* = 0.039) and high (*p* = 0.003) compared with the reference low Lp(a) subgroups. When including only individuals with an initial *V*_max_ of >1.5 m/s, the median rates increased in the high Lp(a) group to 0.05 (−0.04 to 0.20) m/s/year compared to neutral median rates of 0.00 (−0.10 to 0.11) m/s/year in the corresponding low Lp(a) group and 0.00 (−0.04 to 0.13) m/s/year in the intermediate Lp(a) group (*p* = 0.058). As a reference, individuals with ≥2 Doppler measurements but without a Lp(a) measurement (*n* = 9195) had no change in annual median *V*_max_ (0.00 [−0.07 to 0.08] m/s/year) during a median follow‐up of 3.0 years (Fig. 1).

**Fig. 1:**
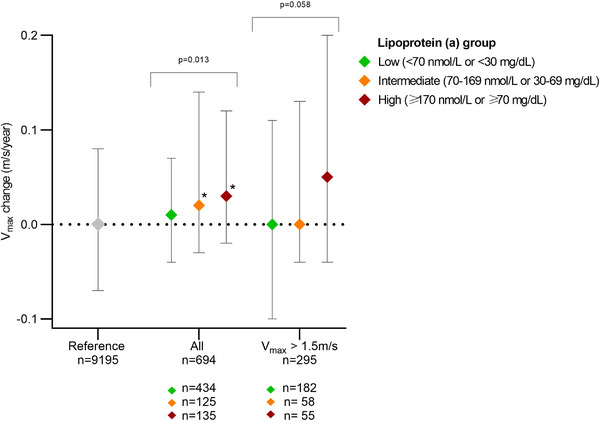
Annual progression rates of peak aortic jet velocities in three lipoprotein(a) (Lp(a)) strata. The annualized progression rates of peak aortic jet velocities, V_max_ change, were calculated for individuals in three strata of Lp(a) based on repeated transthoracic Doppler echocardiography measurements, including all individuals (n = 694) or only individuals with a first V_max_ > 1.5 m/s (n = 295). Individuals in the echocardiography database without an Lp(a) measurement (n = 9195) constituted the reference. p values indicate comparisons of progression rates between Lp(a) strata (Kruskal–Wallis test), and * indicates p < 0.05 compared with the low Lp(a) stratum (Dunn's post hoc test).

This is the first report associating high plasma Lp(a) with a 3‐fold faster *V*_max_ progression compared to low Lp(a) in the general population regardless of the presence or absence of AS at any stage at baseline. Although the *V*_max_ progression was low compared to individuals with mild‐to‐moderate AS [5, 8], the *V*_max_ progression rates are not linear with time. An early small increase in *V*_max_ progression will shorten the time to stages of accelerated *V*_max_ progression towards AS. The present study highlights the long‐term effects of Lp(a) exposure for AS development and identifies Lp(a) as a potential biomarker of AS progression in the general population. These results identify high Lp(a) as a possible indicator of the need for echocardiographic surveillance of AS development. The clinical implications are, however, limited by the fact that, at present, no therapy exists to slow down AS progression [10].

## Author contributions

**Jonas Brinck**, **Karin Littmann** and **Magnus Bäck**: Conceptualization; visualization; investigation; writing; editing and reviewing. **Jonas Brinck** and **Magnus Bäck**: Project administration and funding acquisition. **Linnea Widman**: Methodology/Statistics; reviewing. **Daniel Eriksson Hogling**, **Kenneth Caidahl**, **Maria Eriksson** and **Karolina Szummer**: Reviewing; editing. All authors approve the final version for publication and agree to be accountable for all aspects of the work.

## Conflicts of interest statement

JB reports institutional grants from Amgen, Novartis and Ionis outside the submitted work, and honoraria from Amgen, Novartis, Sanofi and Ultragenyx. DEH has received institutional honoraria from Amgen. KL reports research grant funding from Amgen and Stockholm Innovation Fund outside the submitted work and honoraria from Amgen and Sanofi. MBs’ institution has received speaker and consultant fees from Amarin, Amgen, Heel, Novartis and Fresenius Kabi. The remaining authors have nothing to disclose in relation to this work.

## Funding information

MB is supported by the Swedish Research Council, Grant Number: 2023‐02652; Heart‐Lung Foundation, Grant Number: 20240697.

## Ethics statement

The study was approved by the Ethical Review Authority.

## Supporting information




**Figure S1**. Flowchart for inclusion in cohort study.
**Table S1**. Patient characteristics at the timepoint of the first Doppler measurement.

## Data Availability

The data that support the findings of this study are not publicly available. The study presented here has been subject to an application to an ethical board and approved for publication related to the specific aim of our research project. With reference to the European General Data Protection Regulation (GDPR), the data are personal data and thereby protected by secrecy.
