# Supplementary material for: Elevated lipoprotein(a) and progression of aortic stenosis measured by Doppler echocardiography: A population‐based cohort study
Source: J Intern Med. 2025 May 1;298(1):46–8. doi: 10.1111/joim.20095 (PMC12159711; doi:10.1111/joim.20095)
Supplement: Supplementary file 1 — Figure S1. Flowchart for inclusion in cohort study. Table S1. Patient characteristics at the timepoint of the first Doppler measurement. [file JOIM-298-46-s001.docx]

**Elevated lipoprotein(a) and progression of aortic stenosis measured by Doppler echocardiography: A population-based cohort study**

Jonas Brinck, Karin Littmann, Daniel Eriksson Hogling, Linnea Widman, Kenneth Caidahl, Maria Eriksson, Jonas Johnson, Karolina Szummer and Magnus Bäck

**Supplemental files**

**Figure S1.** Flowchart for inclusion in cohort study

**Table S1.** Patient characteristics at the timepoint of the first Doppler measurement

**Figure S1.** Flowchart for inclusion in cohort study

**
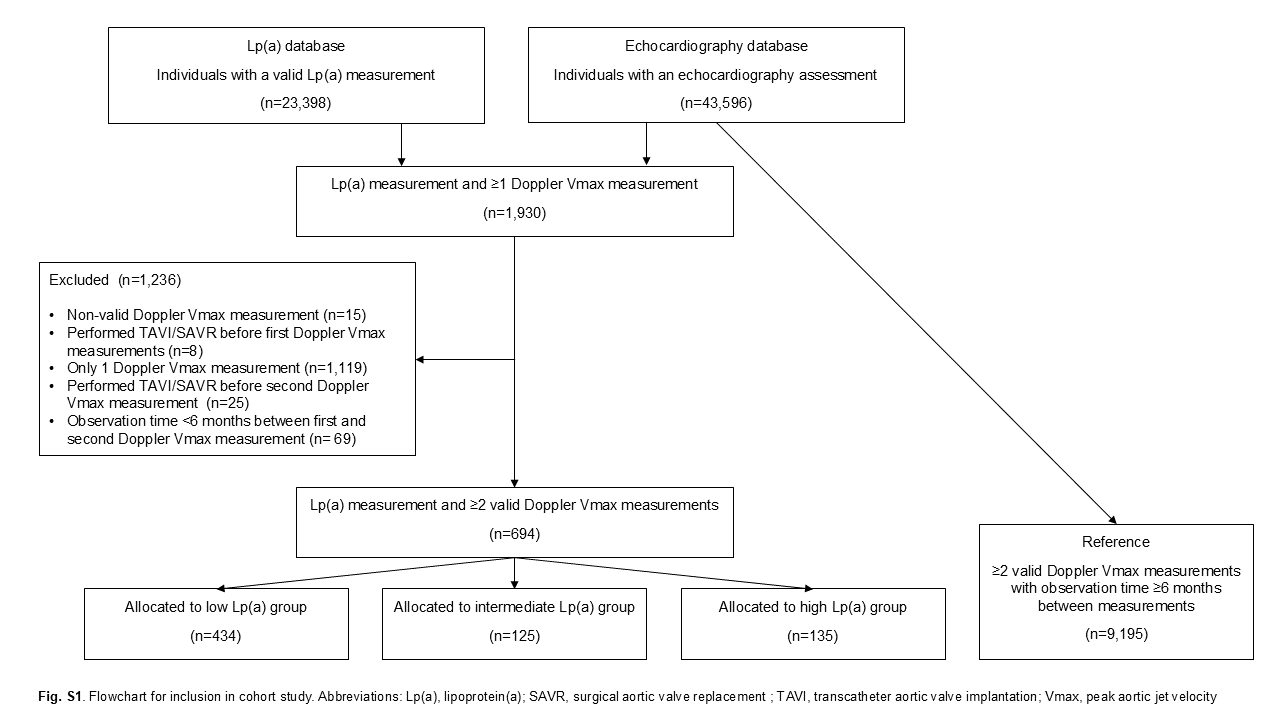
**

**Table S1**. Patient characteristics at the timepoint of the first Doppler measurement

|  | Lp(a) strata | | |  |
| --- | --- | --- | --- | --- |
|  | Low  <70 nmol/L or <30 mg/dL  (n=434) | Intermediate  70-169 nmol/L or 30-69 mg/dL  (n=125) | High  ≥170 nmol/L or ≥70 mg/dL  (n=135) | p-value* |
| Age, years | 51 | 50 | 58 | 0.002 |
| Women, n (%) | 161 (37) | 54 (43) | 52 (39) | 0.47 |
| Median follow-up, years | 3.7 | 2.9 | 3.0 | 0.35 |
| Initial median Vmax, m/s | 1.42 | 1.44 | 1.42 | 0.82 |
| Presence of atherosclerotic cardiovascular disease, n (%) | 102 (23) | 28 (22) | 45 (33) | 0.052 |
| Prior myocardial infarction, n (%) | 35 (8) | 17 (14) | 18 (13) | 0.07 |
| Prior ischemic stroke, n (%) | 20 (5) | 5 (4) | 7 (5) | 0.90 |
| Atrial fibrillation, n (%) | 80 (18) | 26 (21) | 25 (19) | 0.83 |
| Hypertension, n (%) | 228 (53) | 75 (60) | 82 (61) |  |
| Heart failure, n (%) | 57 (13) | 21 (18) | 20 (15) | 0.44 |
| Diabetes, n (%) | 109 (25) | 27 (22) | 32 (24) | 0.71 |
| Pharmacological treatment |  |  |  |  |
| Platelet inhibitor, n (%) | 184 (42) | 48 (38) | 78 (58) | 0.002 |
| Beta blocker, n (%) | 267 (62) | 82 (66) | 87 (64) | 0.64 |
| ACE-inhibitor/ARB, n (%) | 285 (66) | 86 (69) | 92 (68) | 0.75 |
| Calcium antagonist, n (%) | 181 (42) | 71 (57) | 66 (49) | 0.008 |
| Thiazide, n (%) | 234 (54) | 74 (59) | 76 (56) | 0.56 |
| Statin, n (%) | 184 (42) | 58 (46) | 75 (56) | 0.03 |
| Plasma lipid panel** |  |  |  |  |
| Triglycerides, mmol/L (SD) | 1.94 (2.02) | 1.64 (1.08) | 1.63 (0.91) | 0.11 |
| Cholesterol, mmol/L (SD) | 4.63 (1.46) | 4.82 (1.42) | 4.94 (1.17) | 0.08 |
| HDL-cholesterol, mmol/L (SD) | 1.34 (0.47) | 1.40 (0.43) | 1.37 (0.40) | 0.50 |
| LDL-cholesterol, mmol/L (SD) | 2.56 (1.07) | 2.63 (1.08) | 3.09 (0.92) | 0.004 |

*Kruskal-Wallis test or Pearson's chi-squared test
**measured at timepoint when Lp(a) was measured
Abbreviations: ACE, angiotensin converting enzyme; ARB, angiotensin receptor blocker
